# Optimizing the Use of the Gamma-Glutamyl Transpeptidase-to-Platelet Ratio and Transient Elastography to Identify Liver Cirrhosis in Patients with Chronic Hepatitis B Concurrent with Nonalcoholic Fatty Liver Disease

**DOI:** 10.1155/2019/2585409

**Published:** 2019-12-05

**Authors:** Geng-lin Zhang, Shi-cheng Xu, Jie Zeng, Zheng Chen, Yi-Ping Li, Ting Zhang, Zhi-liang Gao

**Affiliations:** ^1^Department of Infectious Diseases, The Third Affiliated Hospital of Sun Yat-sen University, Guangzhou, China; ^2^Guangdong Provincial Key Laboratory of Liver Disease, The Third Affiliated Hospital of Sun Yat-sen University, Guangzhou, China; ^3^Department of Ultrasound, The Third Affiliated Hospital of Sun Yat-sen University, Guangzhou, China; ^4^Department of Stomatology, The Third Affiliated Hospital of Sun Yat-sen University, Guangzhou, China; ^5^Institute of Human Virology and Zhongshan School of Medicine, Sun Yat-sen University, Guangzhou, China

## Abstract

**Background and Aim:**

Little information is available about the assessment and optimal use of the gamma-glutamyl transpeptidase-to-platelet ratio (GPR) and transient elastography (TE) in predicting liver cirrhosis in patients with chronic hepatitis B (CHB) and concurrent nonalcoholic fatty liver disease (NAFLD). This study is aimed at comparing their diagnostic performances and developing an optimal approach for predicting liver cirrhosis in CHB patients with NAFLD.

**Methods:**

Consecutive CHB patients with NAFLD were enrolled. The GPR was calculated, and TE was performed using liver biopsy as a reference standard. The accuracy of predicting liver cirrhosis using GPR and TE was assessed and compared, and an optimal approach was developed.

**Results:**

Both TE and GPR correlated significantly with the histological fibrosis stage. TE and GPR had excellent performance in predicting liver cirrhosis, and the comparison of areas under the receiver operating characteristic curves revealed that TE was superior to GPR (0.95 vs. 0.85, *P* = 0.039). Moreover, the dual cutoffs established by the likelihood ratio showed that GPR had a similar misclassification but higher indeterminate rate than TE (54.5% vs. 11.7%, *P* < 0.001). Additionally, a 2-step approach using GPR followed by TE had comparable performance to that of both GPR and TE tests for all patients (misclassification: 8.9% vs. 8.3%, *P* = 0.866; indeterminate rate: 15.2% vs. 17.2%, *P* = 0.750) but could reduce TE scans by approximately one-third.

**Conclusions:**

Both TE and GPR show excellent performance in predicting liver cirrhosis in CHB patients with NAFLD. The 2-step approach using GPR followed by TE may be optimal for the assessment of cirrhosis in CHB patients with NAFLD.

## 1. Introduction

Hepatitis B virus (HBV) infection continues to be a significant health threat, and an estimated 257 million people are chronically infected [1, 2]. Globally, at least one-third of liver cirrhosis cases are attributable to chronic hepatitis B (CHB), and a significant proportion of CHB patients eventually progress to hepatocellular carcinoma (HCC) [3, 4]. Due to the current obesity epidemic, the rate of nonalcoholic fatty liver disease (NAFLD) in CHB patients is increasing [5]. More importantly, a previous study revealed that concurrent NAFLD independently increased the risk of HCC by 7.3-fold in CHB patients [6]. The presence of liver cirrhosis in particular is an important predictor for overall mortality and liver-related morbidities and mortality [7, 8]. Thus, the early identification of patients with more advanced pathology in a manner that is effective and available for health care systems is urgently needed.

Traditionally, liver biopsy (LB) has been considered the gold standard for the assessment of liver fibrosis and cirrhosis. However, it has disadvantages such as invasiveness, risk of complications, and sampling variability [9]. Therefore, noninvasive approaches have been suggested to overcome these limitations and reduce the need for LB. Previous studies have already demonstrated correlations between fibrosis marker panels and the extent of fibrosis. For example, the aspartate aminotransferase- (AST-) to-platelet ratio index (APRI) and fibrosis-4 (FIB-4) (based on age, AST, alanine aminotransferase (ALT), and platelets) are scores showing satisfactory performance to exclude liver cirrhosis [10]. Recently, the gamma-glutamyl transpeptidase- (GGT-) to-platelet ratio (GPR) has been shown to be more accurate than APRI and FIB-4 in evaluating advanced fibrosis and cirrhosis [11]. Notably, all these markers show less accuracy in identifying intermediate stages of fibrosis. On the other hand, liver stiffness measurement (LSM) measured by transient elastography (TE) is another type of noninvasive method for fibrosis staging and is particularly useful in predicting advanced fibrosis and cirrhosis [12, 13]. However, the existence of NAFLD may cause morphological changes in the liver of CHB patients, which may make TE more difficult to accurately evaluate the degree of fibrosis [13]. Additionally, TE is not widely available in resource-limited areas and adds extra cost.

To our knowledge, little information is available on the assessment and optimal use of TE and GPR in predicting liver cirrhosis in CHB patients with NAFLD. Thus, the chief aim of this study was to (i) compare the diagnostic accuracy of TE and GPR and (ii) develop an optimal approach using TE and GPR in predicting liver cirrhosis in a cohort of CHB patients with NAFLD using histology as a reference.

## 2. Methods

### 2.1. Patients

From July 2013 to September 2018, all treatment-naive Chinese adult CHB patients with NAFLD were enrolled. CHB patients were defined as those who displayed hepatitis B surface antigen (HBsAg) positivity for more than 6 months [14, 15]. NAFLD was diagnosed by the presence of hepatic steatosis (≥5%) on liver biopsy, the absence of significant alcohol consumption (absence is defined as alcohol intake < 30 g/day for men and intake < 20 g/day for women), and the absence of other etiologies that may cause hepatic steatosis [16–18]. The exclusion criteria included the following: receiving antiviral therapy, significant alcohol consumption, coinfection with other hepatitis virus or HIV, concurrent tumors, current use of medications that may cause hepatic steatosis (including corticosteroids, methotrexate, and tamoxifen), and pregnancy. Additionally, patients with body mass index (BMI) ≥ 30 kg/m^2^, AST, or ALT ≥ 5 times the upper limit of normal (ULN) were excluded. Blood samples were obtained on the day of LB examination. Demographic, anthropometric, clinical, and laboratory data were collected. The study was conducted in accordance with the Declaration of Helsinki, and the protocol was approved by our hospital's ethics committee. Informed consent was obtained from each patient.

### 2.2. Liver Histological Analysis

US-guided LB (length > 15 mm) was performed intercostally in the right liver lobe with a 16-gauge or 18-gauge automated edge-cutting biopsy needle (Bard, Tempe, AZ, USA). Liver histology was assessed separately by two experienced pathologists. Both pathologists were blinded to the clinical information and the results of noninvasive tests. Liver fibrosis was staged according to the METAVIR scoring system [19]. Significant fibrosis was defined as stage F2 or higher, whereas cirrhosis was defined as stage F4. Steatosis was defined as the percentage of fat in hepatocytes [17].

### 2.3. LSM Measured by TE

TE was performed with a FibroScan system (Echosens, Paris, France) using the M probe. All LSMs were performed under fasting conditions within 3 days of LB by experienced operators according to the manufacturer's protocol. Operators were blinded to the clinical data and pathology results. The value expressed in kilopascal (kPa) was recorded as a representation of the LSM. Up to 10 valid measurements were performed on each patient. A success rate above 60% and an interquartile range/median ratio of less than 30% were considered reliable [20].

### 2.4. Serum Biomarker Assays

All laboratory parameters were measured by standard automated laboratory methods and using commercially available kits according to the manufacturer's protocols. Complete blood cell counts were determined using an automated hematology analyzer (XE-5000, Sysmex Corporation, Shanghai, China). Biochemical assays were quantitated using a Hitachi 7600 automated biochemistry analyzer (Tokyo, Japan). Coagulation function analyses were measured using an automatic hemostasis/thrombosis analyzer (STA Compact, Holliston, MA, USA). On the basis of these biological parameters, the following noninvasive fibrosis scores were calculated: GPR = [(GGT/upper limit of normal GGT) × 100]/platelet count (10^9^/L) [11]; APRI = [(AST/upper limit of normal AST) × 100]/platelet count (10^9^/L) [21]; and FIB‐4 = [age (years)] × [AST (U/L)]/[platelet count (10^9^/L)] × ALT (U/L)^1/2^ [22].

### 2.5. Virological Analyses

Serum HBV-DNA levels were quantified by Cobas TaqMan (Hoffmann-La Roche, Basel, Switzerland) according to the manufacturer's instructions. HBV serum markers were determined using an Elecsys system (Hoffmann-La Roche, Basel, Switzerland).

### 2.6. Statistical Analysis

Data were expressed as frequencies, medians, and interquartile ranges or means and standard deviations, as appropriate. Differences in variables were analyzed using ANOVA and Student's *t*-tests (for normally distributed data) or the Kruskal–Wallis and Mann–Whitney *U* tests (for nonnormally distributed data), as appropriate. Categorical data were analyzed using a chi-square test and Fisher's exact test. Spearman's correlation was used to analyze the correlations, and the correlation coefficients were compared using Fisher's *Z* test. The diagnostic performance was estimated by using receiver operating characteristic (ROC) curves. The sensitivity, specificity, positive predictive value, negative predictive value, positive likelihood ratio, and negative likelihood ratio were calculated with 95% confidence intervals. Differences between the areas under the ROC curves (AUCs) were compared using DeLong's test. A single cutoff value was determined to achieve a sensitivity of 90% in predicting significant fibrosis or specificity of 90% in predicting cirrhosis. Dual cutoff values for cirrhosis were determined by using multilevel likelihood ratios. Likelihood ratios above 10 and below 0.1 were considered strong evidence to rule in or rule out a diagnosis [23]. A two-tailed *P* < 0.05 was considered statistically significant. Analyses were performed using SPSS version 20.0 (Chicago, IL, USA) and MedCalc version 15.2.2 (MedCalc Software, Mariakerke, Belgium).

## 3. Results

### 3.1. Characteristics of Enrolled Patients

**Figure 1:**
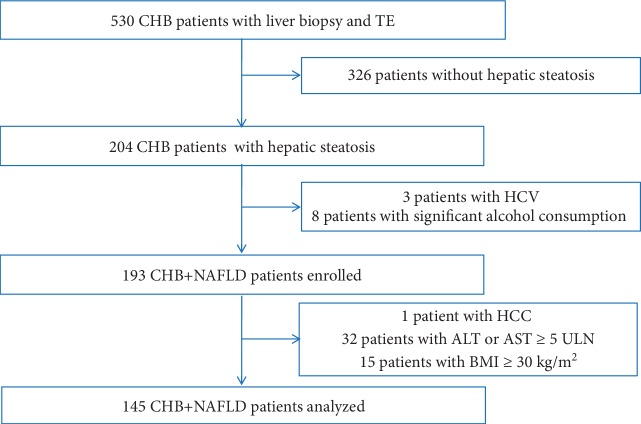
Selection and deposition of patients.

Between July 2013 and September 2018, 530 treatment-naive CHB patients underwent LB and TE scans. Among them, 204 patients had hepatic steatosis. After the exclusion of patients with hepatitis C virus (HCV) infection (*n* = 3) or with significant alcohol consumption (*n* = 8), 193 CHB patients with NAFLD were diagnosed and enrolled. Forty-eight patients were further excluded for the following reasons: 1 patient had concurrent HCC, 32 patients had ALT or AST levels ≥ 5 times of ULN, and 15 patients had BMI ≥ 30 kg/m^2^; thus, 145 patients met the study criteria and were included in the final analysis (Figure 1). Overall, no complications were observed after LB. The median length of LB samples was 17 mm (range, 15-23 mm), and the median portal tract of LB samples was 7 (range, 6-11). Seventy-two (49.7%) patients had significant fibrosis (*F* ≥ 2), and 20 (13.8%) patients had cirrhosis (*F* = 4). The median degree of hepatic steatosis was 10% (range, 5-70%). The main patient characteristics are summarized in Table 1.

### 3.2. Distributions of TE, GPR, APRI, and FIB-4 at Different Fibrosis Stages

**Figure 2:**
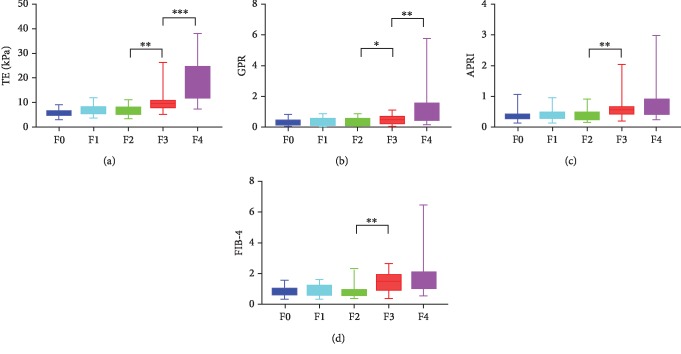
Box and whisker plots of TE, GPR, APRI, and FIB-4 at each fibrosis stage. TE, GPR, APRI, and FIB-4 increased significantly from F2 to F3 (*P* = 0.001, *P* = 0.035, *P* = 0.006, and *P* = 0.001). Moreover, TE and GPR were further increased from F3 to F4 (*P* < 0.001 and *P* = 0.003). However, no differences existed between F3 and F4 for APRI and FIB-4 (*P* = 0.293 and *P* = 0.895). TE: transient elastography; GPR: gamma-glutamyl transpeptidase-to-platelet ratio; APRI: aspartate aminotransferase- (AST-) to-platelet ratio index; FIB-4: fibrosis-4; ^∗^*P* < 0.05; ^∗∗^*P* < 0.01; ^∗∗∗^*P* < 0.001.

The median values of TE, GPR, APRI, and FIB-4 were 7 kPa (range, 3.2-38.5), 0.26 (range, 0.08-5.82), 0.36 (range, 0.12-2.97), and 0.90 (range, 0.25-6.41), respectively. Differences in TE, GPR, APRI, and FIB-4 at different fibrosis stages were analyzed using the Mann–Whitney *U* test. No significant differences were found between F0 and F1 (Z = −0.861, *P* = 0.394; *Z* = −0.151, *P* = 0.883; *Z* = −1.418, *P* = 0.159; and *Z* = −0.697, *P* = 0.492) or between F1 and F2 (*Z* = −1.404, *P* = 0.162; *Z* = −0.899, *P* = 0.372; *Z* = −0.336, *P* = 0.740; and *Z* = −0.704, *P* = 0.487) for TE, GPR, APRI, or FIB-4. Notably, TE, GPR, APRI, and FIB-4 increased significantly from F2 to F3 (*Z* = −3.290, *P* = 0.001; *Z* = −2.100, *P* = 0.035; *Z* = −2.699, *P* = 0.006; and *Z* = −3.289, *P* = 0.001). Moreover, TE and GPR were further increased from F3 to F4 (*Z* = −3.531, *P* < 0.001 and *Z* = −2.922, *P* = 0.003). However, no differences existed between F3 and F4 for APRI and FIB-4 (*Z* = −1.071, *P* = 0.293 and *Z* = −0.146, *P* = 0.895; Figure 2).

### 3.3. Correlations among TE, Serum Biomarkers, Hepatic Steatosis, and Fibrosis Stage

Spearman's correlation was used to analyze the correlations. A strong correlation was found between TE and fibrosis stage (rho = 0.63, *P* < 0.001). Moderate correlations were found between GPR and fibrosis stage (rho = 0.40, *P* < 0.001), APRI and fibrosis stage (rho = 0.39, *P* < 0.001), and FIB-4 and fibrosis stage (rho = 0.38, *P* < 0.001), while the degree of hepatic steatosis did not correlate with TE (rho = 0.024, *P* = 0.772), GPR scores (rho = −0.007, *P* = 0.936), APRI scores (rho = −0.035, *P* = 0.676), or FIB-4 scores (rho = −0.027, *P* = 0.744). Also, no significant differences were found among the correlation coefficients of GPR, APRI, or FIB-4 (all *P* > 0.05). However, the correlation coefficients of TE were significantly higher than those of GPR (*P* = 0.007), APRI (*P* = 0.005), and FIB-4 (*P* = 0.004).

### 3.4. Diagnostic Performance for Significant Fibrosis Assessment

**Figure 3:**
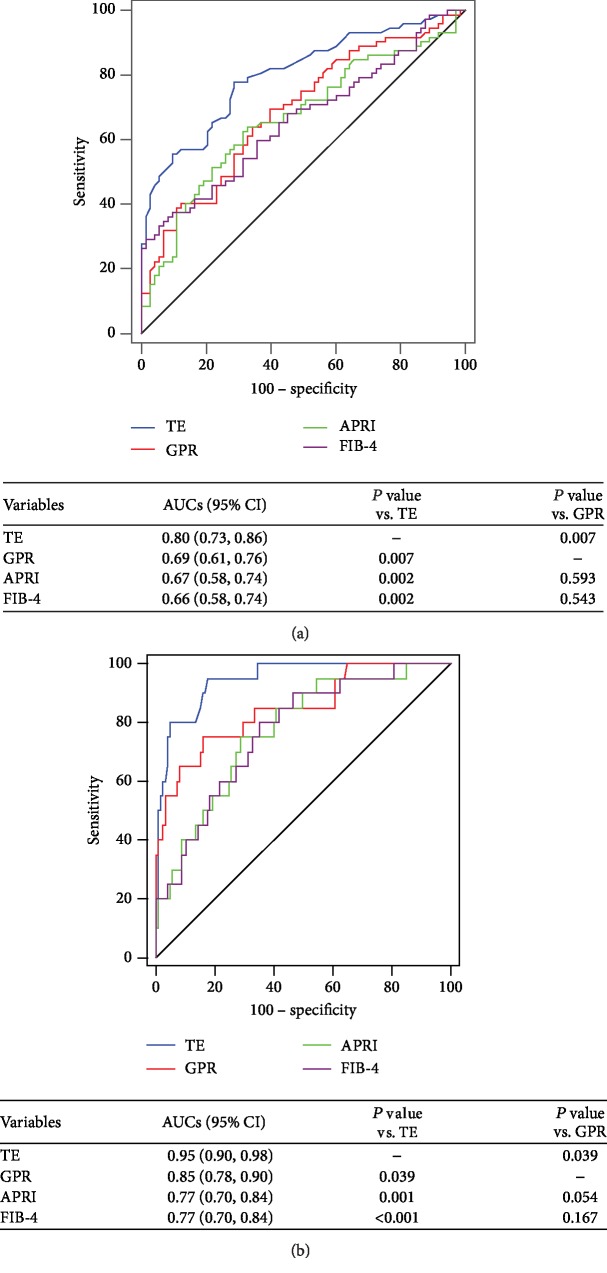
ROC curves of TE, GPR, APRI, and FIB-4 for significant fibrosis assessment (a) and cirrhosis assessment (b) in CHB patients with NAFLD. TE: transient elastography; GPR: gamma-glutamyl transpeptidase-to-platelet ratio; APRI: aspartate aminotransferase- (AST-) to-platelet ratio index; FIB-4: fibrosis-4; AUC: area under the ROC curve; 95% CI: 95% confidence interval.

Figure 3(a) shows the diagnostic performance of TE, GPR, APRI, and FIB-4 for significant fibrosis assessment. The AUCs of TE were higher than those of GPR (0.80 vs. 0.69, *P* = 0.007), APRI (0.80 vs. 0.67, *P* = 0.002), and FIB-4 (0.80 vs. 0.66, *P* = 0.002) for the assessment of significant fibrosis. The desired sensitivity level of 90% was achieved at cutoff values of 5.3 kPa, 0.15, 0.23, and 0.46 for TE, GPR, APRI, and FIB-4, respectively, with corresponding specificities of 38.4%, 27.4%, 13.7%, and 15.1%, respectively (Table 2). Moreover, the specificity of GPR was comparable to that of TE (*P* = 0.062) but higher than that of APRI (*P* = 0.006) and FIB-4 (*P* = 0.016).

### 3.5. Diagnostic Performance for Cirrhosis Assessment

The diagnostic performance of TE, GPR, APRI, and FIB-4 for cirrhosis is shown in Figure 3(b). Table 2 shows the AUCs and predictive values. The comparison of AUCs revealed that TE was significantly superior to serum indexes in predicting cirrhosis (*P* = 0.039, *P* = 0.001, and *P* < 0.001 for GPR, APRI, and FIB-4, respectively; Figure 3(b)). The desired specificity level of 90% for cirrhosis was achieved at cutoff values of 10.7 kPa, 0.56, 0.68, and 1.58 for TE, GPR, APRI, and FIB-4, respectively, with corresponding sensitivities of 80.0%, 65.0%, 40.0%, and 35.0%, respectively (Table 2). The sensitivity of TE was higher than that of GPR (*P* = 0.006), and the sensitivity of GPR was superior to that of APRI and FIB-4 (both *P* < 0.001). In addition, the AUCs of GPR showed a superior trend to APRI and FIB-4 in the assessment of cirrhosis (*P* = 0.054 and *P* = 0.167; Figure 3(b)). Hence, TE and GPR scores were selected for further analysis.

### 3.6. Diagnostic Criteria of TE and GPR in Assessing Cirrhosis

Due to the excellent performance of TE and GPR in predicting liver cirrhosis, a dual cutoff strategy was established by using likelihood ratio analysis to predict liver cirrhosis. The analysis of multilevel likelihood ratios above 10 and below 0.1 was introduced to obtain dual cutoffs for ruling in or ruling out cirrhosis, respectively.  Table 3 shows the performance of TE and GPR for ruling in or ruling out cirrhosis. The results showed that GPR scores lower than 0.21 and values equal to or greater than 0.66 were adequate to rule out and rule in cirrhosis with high diagnostic accuracy (57/66, 86.4%), and GPR values of 0.21 to 0.65 were considered indeterminate. Additionally, TE values lower than 9.0 kPa and values equal to or greater than 10.9 kPa were adequate to rule out and rule in cirrhosis with high diagnostic accuracy (117/128, 91.4%), and TE values of 9.0 kPa to 10.8 kPa were considered indeterminate.

### 3.7. Optimal Use of GPR and TE in Assessing Cirrhosis

**Figure 4:**
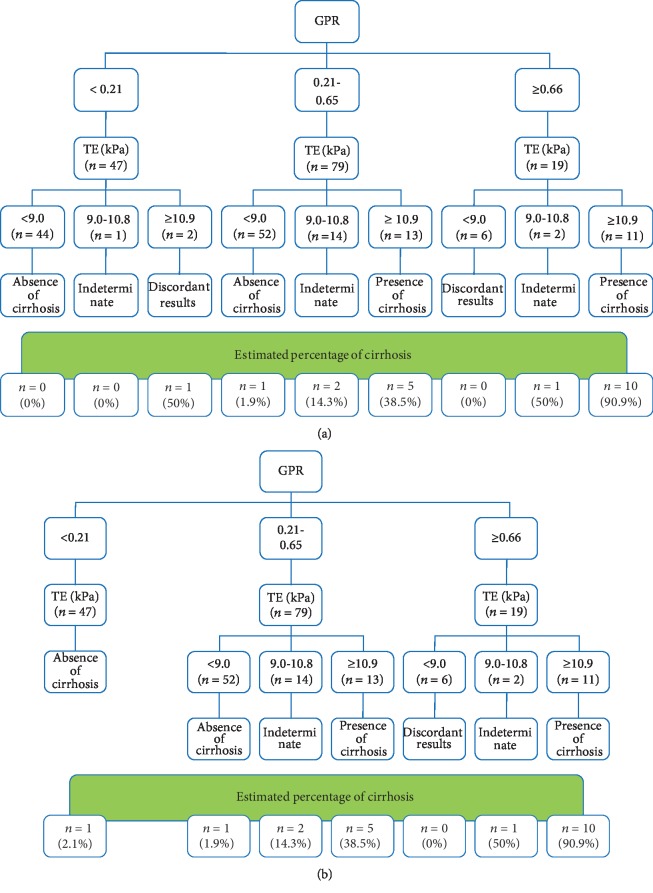
Both GPR and TE tests for all patients (a) and a 2-step approach (GPR followed by TE) (b) for the assessment of liver cirrhosis in CHB patients with NAFLD.

First, the misclassification for using TE alone was similar to that for using GPR alone (8.6% vs. 13.6%, *P* = 0.403). However, the corresponding indeterminate rates of TE were significantly decreased compared with those of GPR (11.7% vs. 54.5%, *P* < 0.001; Table 4). Next, the optimal use of GPR and TE in predicting cirrhosis was explored by two approaches. In the first approach, termed as both tests for all patients, patients were subjected to both GPR and TE (Figure 4(a)). In the second approach, termed as a 2-step approach (GPR followed by TE), patients with GPR < 0.21 were considered to not require TE and not have cirrhosis, while patients with GPR ≥ 0.21 were subjected to a TE scan (Figure 4(b)). Interestingly, the use of the 2-step approach resulted in similar misclassifications (8.9% vs. 8.3%, *P* = 0.866) and comparable indeterminate rates (15.2% vs. 17.2%, *P* = 0.750; Table 4) to that of both tests for all patients. Furthermore, no significant differences in misclassification or indeterminate results were observed among the TE, both test approach and 2-step approach (all *P* > 0.05). These results suggest that TE alone or 2-step approach was useful in predicting cirrhosis, avoiding more than 80% of LB. More importantly, the 2-step approach can further avoid approximately one-third (47/145) of TE scans. Thus, the 2-step approach may be recommended as an optimal strategy for the assessment of liver cirrhosis, especially in TE-limited areas.

## 4. Discussion

**Figure 5:**
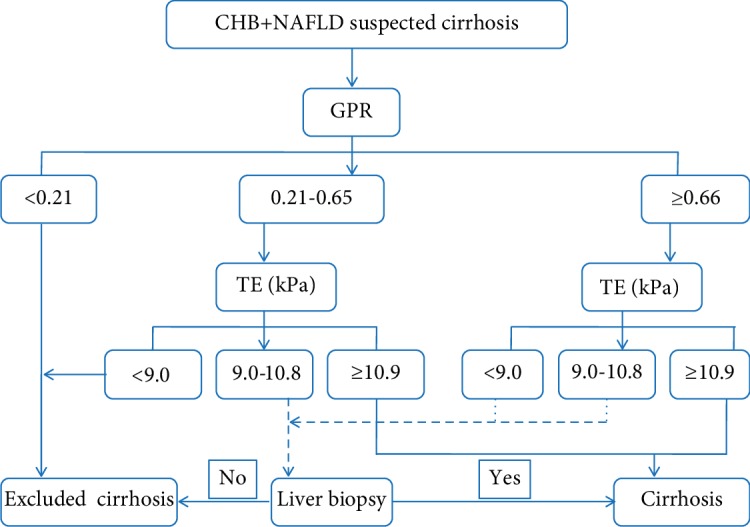
A proposed diagram shows algorithm for the 2-step approach (GPR followed by TE) in the assessment of liver cirrhosis in CHB patients with NAFLD.

This relatively large prospective study focused on CHB patients with NAFLD for the purpose of comparing the diagnostic performance of TE and GPR with histological analysis as a reference and determining an optimal strategy for the evaluation of cirrhosis. To our knowledge, we demonstrate for the first time that both TE and GPR have excellent performance in predicting liver cirrhosis, and TE was superior to GPR (*P* = 0.039). It is also the first to explore the optimal use of GPR and TE in assessing liver cirrhosis. A 2-step approach using GPR followed by TE showed comparable performance to that of both tests but can reduce approximately one-third of TE scans. Moreover, an algorithm using GPR followed by TE in the assessment of liver cirrhosis was proposed (Figure 5).

Fibrosis staging is an essential step in the management of CHB patients to identify those requiring timely treatment. LB has several disadvantages and is very difficult to perform regularly. Thus, noninvasive methods for evaluating liver fibrosis are urgently needed. Several simple biochemical markers, such as APRI and FIB-4, have the advantage of comprising only two or three inexpensive laboratory tests. In 2015, the World Health Organization published its first guideline on the management of CHB. They recommend the use of APRI and FIB-4 as noninvasive tools to predict significant fibrosis and cirrhosis [10]. More recently, a new simple laboratory index termed GPR has been shown to be equivalent or superior to APRI and FIB-4 in CHB patients in several studies [11, 24, 25]. However, less is known about the diagnostic performance of GPR in CHB patients with NAFLD. In this study, GPR increased gradually from F0 to F4 and had a moderate correlation with the fibrosis stage. The performance of GPR in predicting significant fibrosis and cirrhosis was slightly higher than that of APRI and FIB-4, which was different from a recent study reporting that GPR was superior to APRI and FIB-4 in predicting significant fibrosis and GPR was better than FIB-4 in predicting cirrhosis [26]. This variation could be patient-related. Indeed, compared with a previous study, our study recruited fewer HBeAg-positive CHB patients (41.4% vs. 66.4%, *P* < 0.001) and more patients with significant fibrosis (49.7% vs.31.2%, *P* = 0.003). Collectively, GPR showed an acceptable diagnostic performance in predicting liver fibrosis and cirrhosis in CHB patients concurrent with NAFLD. Furthermore, another important strength of GPR lies in its convenience of using routine laboratory index, which does not confer extra costs to the patients.

Although TE may be affected by several factors, it shows good to excellent performance in CHB patients and decreases the need for LB [13, 27]. Similar to our previous study [28], TE had excellent performance in detecting liver fibrosis and cirrhosis, as revealed by AUCs in the current study. To our knowledge, no comparison between TE and GPR in assessing liver fibrosis and cirrhosis in CHB patients with NAFLD has been previously reported. In this study, the overall AUCs of TE for the diagnosis of significant fibrosis and cirrhosis were higher than those of GPR (*P* = 0.007 and *P* = 0.039). These data indicate that TE scan could be a good complement for liver fibrosis staging and have clinical significance in the initiation of antiviral treatment in CHB participants with NAFLD.

Concurrence with NAFLD can increase the risk of HCC development in CHB patients [6]. Thus, it is urgent to identify patients with the stage of cirrhosis earlier. Due to the excellent performance of TE and GPR in evaluating cirrhosis, TE and GPR might be simple and readily available noninvasive methods for detecting this condition. To determine a practical diagnostic cutoff value, a dual cutoff strategy established by likelihood ratio analysis was used [23]. Our results showed that TE values ≥ 10.9 kPa or GPR values ≥ 0.66 indicated a high risk of cirrhosis and may indicate a prioritization for antiviral treatment. In contrast, TE values < 9.0 kPa or GPR values < 0.21 may indicate a low risk of cirrhosis. Furthermore, the misclassification when using GPR alone was similar to that when using TE alone. However, the corresponding indeterminate results were significantly higher when using GPR alone than using TE alone (54.5% vs. 11.7%, *P* < 0.001). These findings indicate that GPR, easy to use and inexpensive, may be used for preliminary evaluation, and TE scan could be used for further confirmation. Then, the optimal use of GPR and TE in predicting liver cirrhosis was explored by two approaches. And interestingly, the 2-step approach had comparable performance to that of both tests for all patients (Table 4). Moreover, approximately one-third of TE scans can be avoided when using the 2-step approach, which can further reduce the patient's cost. Collectively, patients with low GPR are unlikely to have cirrhosis and may be followed with the test regularly. On the other hand, patients with indeterminate or high GPR should be referred to a TE scan for further confirmation (Figure 5). This stepwise strategy would be available and valuable for the noninvasive evaluation of liver cirrhosis in CHB patients with NAFLD, especially in resource-limited areas.

To date, our study is the largest one focusing on biopsy-proven CHB patients with NAFLD for the assessment of liver cirrhosis. A novel optimal approach using GPR and TE was proposed for the assessment of liver cirrhosis in CHB patients with NAFLD. However, this study has several limitations. First, as the controlled attenuation parameter was unavailable in our department until 2018, it was not involved in the comparison. Additionally, patients with BMI ≥ 30 kg/m^2^ were excluded due to the unavailability of XL probe for TE, which may induce a bias. Moreover, although effective in this study, the 2-step approach is not the final word but rather a suggestion that requires further validation.

In conclusion, both GPR and TE show excellent performance in evaluating liver cirrhosis in CHB patients with NAFLD. A novel algorithm using the 2-step approach (GPR followed by TE) could be an optimal approach for the assessment of liver cirrhosis.

## Figures and Tables

**Table 1:** Patients' characteristics.

Characteristic	Standard value (range)	Patients (*n* = 145)
Age (years)	NA	37.77 ± 8.28
Male gender (*n*, %)	NA	123 (84.8%)
Body mass index (BMI) (kg/m^2^)	NA	24.14 ± 2.92
Aspartate aminotransferase (U/L)	15-40	29 (25-36)
Alanine aminotransferase (U/L)	3-35	40 (29-58.5)
Total bilirubin (*μ*mol/L)	4-23.9	12.9 (10.7-16.9)
Albumin (g/L)	36-51	44.54 ± 3.22
Gamma-glutamyl transpeptidase (U/L)	10-60	32 (22-46)
Platelets count (10^3^/mm^3^)	100–350	199.48 ± 54.62
Prothrombin time activity (%)	70–120	96.28 ± 11.06
Fasting glucose (mmol/L)	3.9-6.1	4.94 (4.64-5.37)
Total cholesterol (mmol/L)	3.1-5.7	4.84 (4.32-5.22)
Triglyceride (mmol/L)	0.34-1.92	1.21 (0.95-1.49)
HBeAg positive (*n*, %)	>1	60 (41.4%)
HBV-DNA (log_10_ IU/mL)	<20 IU/mL	4.96 (3.68-6.71)^†^
Fibrosis score (METAVIR)		
F0 (*n*, %)	NA	25 (17.2%)
F1 (*n*, %)	NA	48 (33.1%)
F2 (*n*, %)	NA	29 (20.0%)
F3 (n, %)	NA	23 (15.9%)
F4 (*n*, %)	NA	20 (13.8%)
Hepatic steatosis ≥5%	NA	62 (42.8%)
10-19%	NA	42 (29.0%)
≥20%	NA	41 (28.2%)

Data were expressed as means and standard deviations or medians and interquartile ranges (25^th^-75^th^). ^†^Eighteen patients had undetectable HBV-DNA levels. NA: not applicable.

**Table 2:** Diagnostic performance of TE, GPR, APRI, and FIB-4 in CHB patients with NAFLD for significant fibrosis and cirrhosis assessment.

Variables	TE	GPR	APRI	FIB-4
Significant fibrosis assessment (*F* ≥ 2)				
Cutoff value	5.3 kPa	0.15	0.23	0.46
AUC	0.80 (0.73, 0.86)	0.69 (0.61, 0.76)	0.67 (0.58, 0.74)	0.66 (0.58, 0.74)
Sensitivity (%)	90.3 (81.0, 96.0)	90.3 (81.0, 96.0)	90.3 (81.0, 96.0)	90.3 (81.0, 96.0)
Specificity (%)	38.4 (27.2, 50.5)	27.4 (17.6, 39.1)	13.7 (6.8, 23.8)	15.1 (7.8, 25.4)
PPV (%)	59.1 (49.3, 68.4)	55.1 (45.7, 64.3)	50.8 (41.8, 59.7)	51.2 (42.2, 60.1)
NPV (%)	80.0 (63.1, 91.6)	74.1 (53.7, 89.9)	58.8 (32.9, 81.6)	61.1 (35.7, 82.7)
Positive LR	1.46 (1.2, 1.8)	1.24 (1.1, 1.5)	1.05 (0.9, 1.2)	1.06 (0.9, 1.2)
Negative LR	0.25 (0.1, 0.5)	0.35 (0.2, 0.8)	0.71 (0.3, 1.8)	0.65 (0.3, 1.6)

Cirrhosis assessment (*F* = 4)				
Cutoff value	10.7 kPa	0.56	0.68	1.58
AUC	0.95 (0.90, 0.98)	0.85 (0.78, 0.90)	0.77 (0.70, 0.84)	0.77 (0.70, 0.84)
Sensitivity (%)	80.0 (56.3, 94.3)	65.0 (40.8, 84.6)	40.0 (19.1, 63.9)	35.0 (15.4, 59.2)
Specificity (%)	91.2 (84.8, 95.5)	90.4 (83.8, 94.9)	90.4 (83.8, 94.9)	90.4 (83.8, 94.9)
PPV (%)	59.3 (38.8, 77.6)	52.0 (31.3, 72.2)	40.0 (19.1, 63.9)	36.8 (16.3, 61.6)
NPV (%)	96.6 (91.5, 99.1)	94.2 (88.4, 97.6)	90.4 (83.8, 94.9)	89.7 (83.0, 94.4)
Positive LR	9.09 (5.0, 16.7)	6.77 (3.6, 12.7)	4.17 (1.9, 8.9)	3.65 (1.6, 8.1)
Negative LR	0.22 (0.09, 0.5)	0.39 (0.2, 0.7)	0.66 (0.5, 1.0)	0.72 (0.5, 1.0)

The cutoff value was determined to achieve a sensitivity of 90% in predicting significant fibrosis and a specificity of 90% in predicting cirrhosis. Data in parentheses were 95% confidence interval. TE: transient elastography; GPR: gamma-glutamyl transpeptidase-to-platelet ratio; APRI: aspartate aminotransferase- (AST-) to-platelet ratio index; FIB-4: fibrosis-4; AUC: area under the ROC curve; PPV: positive predictive value; NPV: negative predictive value; LR: likelihood ratio.

**Table 3:** Performance of TE and GPR for ruling in or ruling out cirrhosis using liver biopsy as a reference standard.

Variables	GPR (ruling out)	GPR (ruling in)	TE (ruling out)	TE (ruling in)
Cutoff value	0.21	0.66	9.0 kPa	10.9 kPa
Sensitivity (%)	95.0 (75.1, 99.9)	55.0 (31.5, 76.9)	95.0 (75.1, 99.9)	80.0 (56.3, 94.3)
Specificity (%)	37.6 (29.1, 46.7)	94.4 (88.8, 97.7)	82.4 (74.6, 88.6)	93.6 (87.8, 97.2)
PPV (%)	19.6 (12.2, 28.9)	61.1 (35.7, 82.7)	46.3 (30.7, 62.6)	66.7 (44.7, 84.4)
NPV (%)	97.9 (88.9, 99.9)	92.9 (87.0, 96.7)	99.0 (94.8, 100)	96.7 (91.8, 99.1)
Positive LR	1.52 (1.3, 1.8)	9.82 (4.3, 22.3)	5.40 (3.6, 8.0)	10.00 (5.3, 18.8)
Negative LR	0.13 (0.02, 0.9)	0.48 (0.3, 0.8)	0.061 (0.01, 0.4)	0.21 (0.09, 0.5)

The dual cutoff values for cirrhosis were determined by using multilevel likelihood ratios. Likelihood ratios above 10 and below 0.1 were considered strong evidence to rule in or rule out liver cirrhosis. Data in parentheses were 95% confidence interval. TE: transient elastography; GPR: gamma-glutamyl transpeptidase-to-platelet ratio; PPV: positive predictive value; NPV: negative predictive value; LR: likelihood ratio.

**Table 4:** Performance of GPR alone, TE alone, both tests for all patients, and the 2-step approach in the assessment of cirrhosis.

Variables	GPR alone	TE alone	Both tests for all patients	2-step approach	*P* value (both tests vs. 2-step approach)
Sensitivity, % (*n*/*N*)	91.7 (11/12)	94.1 (16/17)	93.8 (15/16)	88.2 (15/17)	0.963
Specificity, % (*n*/*N*)	85.2 (46/54)	91.0 (101/111)	91.3 (95/104)	91.5 (97/106)	0.846
PPV, % (*n*/*N*)	57.9 (11/19)	61.5 (16/26)	62.5 (15/24)	62.5 (15/24)	1.000
NPV, % (*n*/*N*)	97.9 (46/47)	99.0 (101/102)	99.0 (95/96)	98.0 (97/99)	0.988
Positive LR	6.19	10.45	10.83	10.39	—
Negative LR	0.10	0.06	0.07	0.13	—
Misclassification, % (*n*/*N*)	13.6 (9/66)	8.6 (11/128)	8.3 (10/120)	8.9 (11/123)	0.866
Indeterminate, % (*n*/*N*)	54.5 (79/145)	11.7 (17/145)	17.2 (25/145)	15.2 (22/145)	0.750
Accuracy, % (*n*/*N*)	86.4 (57/66)	91.4 (117/128)	91.7 (110/120)	91.1 (112/123)	0.866

TE: transient elastography; GPR: gamma-glutamyl transpeptidase-to-platelet ratio; PPV: positive predictive value; NPV: negative predictive value; LR: likelihood ratio.

## Data Availability

The data used or analyzed during the current study are available from the first author (Geng-lin Zhang, email: zhanggenglin1984@163.com) on reasonable request.

## Abbreviations

CHB: Chronic hepatitis B
NAFLD: Nonalcoholic fatty liver disease
LB: Liver biopsy
TE: Transient elastography
GPR: Gamma-glutamyl transpeptidase-to-platelet ratio
APRI: Aspartate aminotransferase-to-platelet ratio index
FIB-4: Fibrosis-4
ROC curve: Receiver operating characteristic curve
AUCs: Areas under the ROC curves.
